# Evaluation of the applicability of hospital-affiliated green spaces to patient recovery using the entropy weight method and grey relational analysis

**DOI:** 10.3389/fpubh.2024.1362884

**Published:** 2024-06-13

**Authors:** Wei Yuan, Maopeng Yang, Dan Liu, Xingyuan Fu, Lei Yu, Kun Wang

**Affiliations:** ^1^College of Horticulture & Landscape Architecture/Art Academy, Northeast Agricultural University, Harbin, China; ^2^Respiratory Oncology Department, Harbin Medical University Cancer Hospital, Harbin, China

**Keywords:** hospital affiliated green space, landscape evaluation, evaluation of patient recovery applicability, entropy weight method (EWM), grey relational analysis (GRA)

## Abstract

**Introduction:**

Hospital affiliated green spaces can help patients recover and recover their physical functions, promote physical and mental relaxation, enhance health awareness, and improve overall health. However, there are still significant questions about how to scientifically construct hospital affiliated green spaces. This study examines the impact of hospital green spaces on patient rehabilitation through scientific evaluation methods, providing reference for the scientific construction of hospital affiliated green spaces. Applicability evaluation was conducted on the affiliated green spaces of three hospitals in Harbin. An evaluation system covering plants, space, accessibility, rehabilitation functions, and promotional and educational functions has been constructed. The entropy weight method is used to determine the weight of indicators, and the grey correlation analysis method is used to evaluate the suitability of green space for patient rehabilitation.

**Methods:**

The experimental results showed that the landscape accessibility index had the highest weight (0.3005) and the plant index had the lowest weight (0.1628), indicating that caring for special needs is the foundation of hospital landscapes, and plants have subtle and long-term effects on physical and mental health. In the evaluation of the rehabilitation applicability of the affiliated green spaces of various hospitals, the second hospital has the highest grey correlation degree (0.8525), followed by the tumor hospital (0.5306) and the fifth hospital (0.4846). It can be seen that the green space of the second hospital has high applicability for patient rehabilitation, but the green space of the tumor hospital and the fifth hospital needs to be improved and developed.

**Results and discussion:**

The evaluation criteria used in this study are comprehensive. The landscaping at the Third Hospital is well-planned with good plant configuration and reasonable spatial layout. However, there is insufficient consideration for accessibility in the landscape design, and the details are lacking. The rehabilitation and educational functions of the landscape are inadequate, with limited outdoor activities and low road safety. The hospital′s affiliated green spaces should adhere to the principle of “appropriate scale, comprehensive functionality, and educational leisure,” integrating rehabilitation and educational functions while increasing the variety of outdoor activities. In the future, emphasis should be placed on exploring the integration of landscape and rehabilitation to provide a functional site that is convenient for visiting, with improved rehabilitation facilities and an educational and enjoyable environment. The design should incorporate elements that contribute to a sense of well-being, including roads and

## 1 Introduction

As a medical ‘soft environment’, hospital affiliated green space is a landscape for auxiliary healing. High-quality hospital affiliated green space can increase the pleasure and knowledge of the target population, thereby serving patients’ recovery and increasing the social value of the hospital. In the 1980s, environmental behaviorist Dr. Ulrich proposed the hypothesis of natural benefits to humans based on elements such as water, green plants, and vegetation in natural landscapes. He believed that humans have an instinctive preference response to all natural environments, which not only has a positive effect on physiology but also alleviates their own stress. Dr. Ulrich’s full study on the role of plant landscapes in the postoperative recovery of gallbladder patients was published in *Science* (1). The study showed that appreciating natural landscapes can help patients recover faster and use fewer painkillers. In the early stages of theoretical research, scholars used data comparison, a range of measurement instruments and statistical scales and reviews of the literature to verify that the natural environment, more than the urban environment, has a positive impact on pain relief, recovery from stress and attention (2–8). They also demonstrated clearly the benefits of purposeful integration of the natural landscape into patient treatment (9–11).

As research progresses, scholars have begun to focus on healing gardens for specific conditions, age groups and even treatment phases (12–20). It is believed that, given the differences in patients’ ages and symptoms, landscape design should be patient-led and should seek to design more targeted garden landscapes to improve the healing efficiency of the landscape (21–23). The construction of hospital healing gardens has been shown not only to be beneficial for the physical and mental rehabilitation of patients but also to play a positive role in relieving pressure on medical personnel and the family members of patients (24, 25). Accordingly, scholars have begun to explore the mechanism and development of healing processes in healing gardens (26), to compare research processes in relation to healing landscape theory in different countries (27) and to emphasise the conservation of biodiversity in healing gardens (28).

According to the research summarised above, hospital affiliated green space should be a caring landscape that assists rehabilitation and the recovery of physical function, facilitates mental and physical relaxation, enhances health awareness and improves overall levels of health. However, despite an increase in the number of studies on hospital green space landscapes, the bias toward landscape design strategy has continued. Research on the evaluation of hospital green space landscape remains relatively scarce and is characterised by a number of shortcomings. The Analytic Hierarchy Process (AHP) refers to the systematic approach of decomposing a complex multi-objective decision-making problem into multiple objectives or criteria, and then into several levels of multiple indicators (or criteria, constraints). By using qualitative indicator fuzzy quantification methods, the hierarchical single ranking (weight) and total ranking are calculated, which serve as the objective (multiple indicators) and multi scheme optimization decision-making system. However, due to the limited quantitative data and numerous qualitative components of AHP, it is not easily convincing. As most relevant evaluation studies use the subjective analytic hierarchy process (AHP) (29–32), objectivity is neglected, evaluation systems are not robust and adaptability to practical problems is low. Moreover, when there are too many indicators, the data statistics are excessive. In addition, evaluation indexes focus on the basic needs of landscape construction and user comfort (20, 33) and pay little attention to the rehabilitation function of the landscape. Nonetheless, with scientific developments and improvements in people’s awareness, demand for convalescent green space functions has continued to deepen and refine. As an indirect medical method, rehabilitation plays a role in the functional trend for healing resources in hospital green space. More work is required to determine how to improve the patient recovery applicability of the affiliated green spaces of modern hospitals through landscape means, to explore the integration of landscape and rehabilitation and to provide the target population with functional plots that are easy to visit, complete in terms of rehabilitation function and entertaining in terms of education. Scientific systems of evaluation must be used to clarify both the problem orientation and the method orientation in hospital landscape design.

The entropy weight method (EWM) is a mathematical approach that assigns weights based on the degree of variation (coefficient of difference) of evaluation indicators. Advantages of the technique include high accuracy and adaptability, and the weight results are more objective compared to AHP. EWM is widely used; for example, it has been used by Liu et al. (34) to construct a comprehensive index of antioxidant enzymes in Tan mutton, and by Qin et al. (35) to reveal the corrosion resistance of fibre-reinforced concrete to sodium sulphate solution under different conditions. Grey relational analysis (GRA) is a method of measuring the degree of correlation between factors based on the similarity or dissimilarity of their development trends. The approach has a strong tolerance for sample patterns and low computational complexity, and can accurately rank the correlation between multiple indicator data and their ideal values. Mausam et al. (36), for instance, validated and optimised experimental data and results using GRA in their research on converting solar energy into usable thermal energy. The present study, which follows the principle of ‘appropriate scale, perfect function, entertaining’, combines EWM with GRA to give a comprehensive evaluation of the affiliated green space of three hospitals in Harbin. The purpose is to clarify the full potential of hospital affiliated green space in patient recovery. The results provide a scientific basis for improving the healing function of hospital affiliated green space, and for increasing the use value and rehabilitation value of that landscape. The study also has significant practical implications for the construction of medical software and the hardware environment.

## 2 Materials and methods

### 2.1 Study area

Second Affiliated Hospital of Harbin Medical University, established in 1954 and located in Nangang District, Harbin City, Heilongjiang Province (45°69′53″N, 126°62′07″E), has an area of 50 hm^2^. It is a large-scale tertiary general hospital that covers the largest area of any hospital in Harbin.

Fifth Hospital of Harbin, established in 1955 and located in Xiangfang District, Harbin City (45°70′88″N, 126°65′68″E), has an area of 5 hm^2^. It is a general tertiary hospital that focuses on orthopaedics and burns injuries. The Ministry of Health lists it as the network hospital of the National Emergency Rescue Centre.

**Figure 1:**
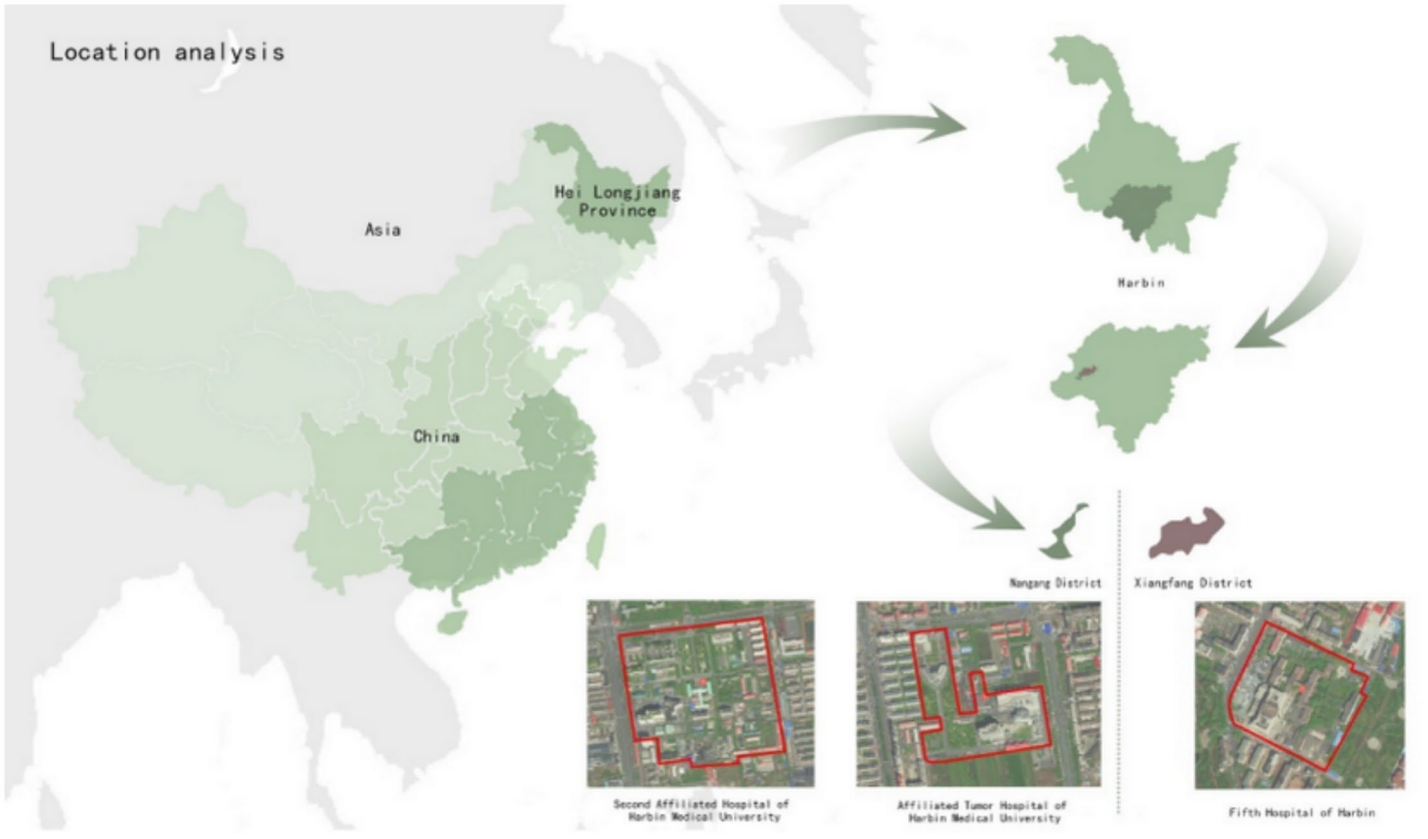
Location analysis.

Affiliated Tumour Hospital of Harbin Medical University, established in 1972 and located in Nangang District, Harbin City (45°69′73″N, 126°64′01″E), has an area of 24 hm^2^. It is a general tertiary hospital that integrates tumour prevention, medical treatment, teaching and scientific research (Figure 1).

Each of the three hospitals provides green space for visiting and resting. The green landscape of Second Hospital is intended to assist in the rehabilitation of patients at all levels. Fifth Hospital gives priority to patients being treated for burns, and Tumour Hospital gives priority to patients with tumour-related diseases; thus, the service-oriented goals of their green spaces affiliated are clear. All three hospitals are suitable sites for comprehensive research on the landscape and rehabilitation application of hospital affiliated green spaces.

All three institutions are tertiary hospitals that integrate medical treatment, scientific research and teaching. They all have a certain proportion of rehabilitation green spaces for patients to visit and relax in, and thus they represent high landscape research value. However, there are also some differences between them.

Second Affiliated Hospital of Harbin Medical University is a comprehensive organisation, with a variety of plant species in its green space, and a good combination of trees, shrubs and grasses. The terrain is flat, the spatial functions are diverse and the sense of scale is reasonable. There are many activity facilities, and garden areas emphasise artistic care. The form of education is relatively single, and the types of patients are diverse and numerous.

Fifth Hospital of Harbin focuses on orthopaedic and burn patients. Its green spaces mainly consist of trees, with fewer shrubs and less ground cover. The forest is open, with large spaces and single functions. The boundary between functional spaces is weak, and the overall terrain is flat with small height differences. The accessibility of facilities is high.

Finally, Affiliated Tumour Hospital of Harbin Medical University focuses on cancer patients. Its green spaces are mainly dominated by shrubs, with sparse trees, a simple spatial layout, flat terrain and few rehabilitation facilities. However, its garden areas have strong artistic qualities.

### 2.2 Construction of the evaluation index system

In terms of applicability to patient recovery, factors in the evaluation of hospital affiliated green space include, but are not limited to, space, safety and rehabilitation function. A complex evaluation system is therefore required, and its construction must objectively and reasonably reflect the advantages and disadvantages of the current hospital-affiliated green space landscape. By comparing, analysing and generalising from the relevant literature on the evaluation of healing landscapes, the present study selected plant configuration, spatial diversity and comfort of facilities as its factors for evaluation. The evaluation system was initially constructed to cover five aspects: landscape planting, space, accessibility, rehabilitation function and publicity and education function (37–39). Plant configuration directly affects the spatial and landscape aesthetics of plants, as well as the impact of landscape on rehabilitation, and is the foundation of these three. Plants have diverse colours and different forms in different seasons, and selecting and matching plants can enhance the beauty of the landscape, leading to a positive and optimistic mood. Therefore, plant colours and seasonal changes can be used as evaluation factors. Plants can be coordinated with surrounding buildings to form a unified environmental colour, which can make people feel more comfortable and relaxed. Green space refers to the environment covered by vegetation within an urban planning area. Diverse spaces and reasonable spatial scales and layouts can enhance the effectiveness and quality of green landscapes, forming beautiful and functional green spaces. Accessibility means that green landscapes are free from obstacles and hazards. These landscapes should have the potential to facilitate rehabilitation by stimulating patients’ senses. The popularisation of science and education is an important function of green landscapes and the main way to display green landscape. A sound popularisation of science and education function is the key to the effectiveness of wetland parks.

In the present study, the index factors were screened, adjusted and supplemented through field investigation, based on relevant literature (39) and feedback from experts in related fields in three rounds of discussion. The final evaluation index system was established by applying the three levels of target, criteria and index. The target layer is the evaluation of the applicability of hospital-affiliated green space landscape to patient recovery. The criterion layer is based on the principle of ‘appropriate scale, perfect function, entertaining’ that hospital affiliated green space should follow, and on the basic requirements of landscape construction, such as plants and facilities. The index layer consists of the corresponding index factors and forms the final evaluation system (Table 1).

**Table 1:** Evaluation system for the applicability of hospital affiliated green space to patient recovery.

Target hierarchy (A)	Criterion layer (B)	Index layer (C)
Evaluation of patient recovery applicability of hospital affiliated green space landscape (A)		Configuration of plants (C1)
	Plants (B1)	Plant colours and seasonal change (C2)
		Harmony between plants and the environment (C3)
		Spatial diversity (C4)
	Space (B2)	Rationality of spatial scale (C5)
		Rationalisation of spatial layout (C6)
		Comfort of facilities (C7)
	Accessibility (B3)	Road safety (C8)
		Information accessibility of facilities (C9)
	Rehabilitation function (B4)	Range of activities (C10)
		Facilities participation (C11)
		Caring landscape art (C12)
	Publicity and education function (B5)	Ornamental value (C13)
		Degree of propaganda (C14)
		Interactivity (C15)

### 2.3 Index scoring criteria

The selection of scoring standard values in this study was carried out (1) in line with the corresponding laws and regulations, (2) with reference to industry-related standards (ISO 11091:1994) and related literature in the research field (40), (3) with regard to clear and reasonable index scoring criteria and (4) incorporating analysis of field survey results.

### 2.4 Comprehensive calculation model

#### 2.4.1 Comprehensive calculation model for the entropy weight method (EWM)

EWM was used to determine the weight of each index in the evaluation system. First, according to mathematical principles, a questionnaire was designed to score the importance of each evaluation factor in the index layer on a scale from 1 to 5 points.

Setting the target layer (A) of the evaluation system to *X*, the evaluation factors of the criterion layer were numbered $I_{1},I_{2},\cdots ,I_{i}$ , the evaluation factors of the index layer were numbered $I_{11},I_{12},I_{13},\cdots ,I_{i1},I_{i2},I_{i3},\cdots ,I_{ij}$ , and the evaluation number of each score of the index layer $I_{ij}$ was set to $x_{ijl}$ , where $P\left(x_{ijl}\right)$ is the proportion of $x_{ijl}$ in the total evaluation number. If $x_{ijl}$ = 0, it is not included in the calculation.

The proportion of $x_{ijl}$ included in the calculation of the total evaluation number $P\left(x_{ijl}\right)$ is:


(1) $$P_{\left(x_{ijl}\right)}=\frac{x_{ijl}}{\sum_{i=1}^{bn}x_{ijl}}$$

where *b* is the number of evaluation factors of the index layer $I_{ij}$ under criterion layer $I_{i}$ , and *n* is the number of evaluation factors of criterion layer $I_{i}$ under the target layer *X*.

The entropy value of each factor in the index layer of the evaluation index system $H_{\left(xij\right)}$ is:


(2) $$H_{\left(xij\right)}=-K\overset{5}{\underset{l=1}{\sum }}P_{\left(x_{ijl}\right)}\ln P_{\left(x_{ijl}\right)}$$

where 5 represents the five grades, $k=\frac{1}{\ln n}$ , and $H_{\left(xij\right)}\in \left[0,1\right]$ .

The entropy weight of each factor in the index layer $w_{ij}$ is:


(3) $$w_{ij}=\frac{1-H_{\left(xij\right)}}{b-\sum_{j=1}^{b}H_{\left(\mathrm{xijl}\right)}}$$

where $w_{ij}\in \left[0,1\right]$ , $\overset{m}{\underset{j=1}{\sum }}w_{ij}=1$ , and *m* is the total number of evaluation factors in the index layer.

The entropy values of each factor in the criterion layer $H_{\left(x_{i}\right)}$ is:


(4) $$H_{\left(x_{i}\right)}=\overset{b}{\underset{j=1}{\sum }}w_{ij}\cdot H_{\left(xij\right)}$$

The entropy weight of each factor in the criterion layer $w_{i}$ is:


(5) $$w_{i}=\frac{1-H_{\left(x_{i}\right)}}{n-\sum_{i=1}^{n}H_{\left(x_{i}\right)}}$$

The comprehensive weight of evaluation factors in the index layer $W_{ij}$ is:


(6) $$W_{\left(ij\right)}=w_{ij}w_{i}$$

The comprehensive weight of evaluation factors in the criterion layer $W_{i}$ is:


(7) $$W_{i}=\overset{b}{\underset{j=1}{\sum }}W_{ij}$$

Thus, the comprehensive weight of the index layer evaluation factor $W_{ij}$ and the comprehensive weight of the criterion layer evaluation factor $W_{i}$ can be calculated.

#### 2.4.2 Comprehensive evaluation model for grey relational analysis (GRA)

GRA was used to evaluate the landscape of hospital rehabilitation green space. The questionnaire was designed to score the level of satisfaction for each factor in the index layer on a scale from 1 to 100 points. After sorting the questionnaire results, the scores of each factor in the index layer formed the following matrix:


$$\left(x{'}_{1},x{'}_{2},\cdots ,x{'}_{n}\right)=\left(\begin{array}{cccc} x{'}_{1}\left(1\right) & x{'}_{2}\left(1\right) & \cdots & x{'}_{n}\left(1\right)\\ x{'}_{1}\left(2\right) & x{'}_{2}\left(2\right) & \cdots & x{'}_{n}\left(2\right)\\ \vdots & \vdots & & \vdots \\ x{'}_{1}\left(m\right) & x{'}_{2}\left(m\right) & \cdots & x{'}_{n}\left(m\right) \end{array}\right)$$

where *m* is the number of evaluation factors in the index layer, and *n* is the number of evaluation factors in the criterion layer.

The dimensionless processing of the evaluation index data resulted in the dimensionless value of *xi*:


(8) $$x_{i}=\frac{x{'}_{i}-x{'}_{i\min}}{x{'}_{i\max}-x{'}_{i\min}}$$

where $x{'}_{i}$ is the corresponding score of the evaluation factor, $x{'}_{i\max}$ is the maximum value of the corresponding score of the evaluation factor, and $x{'}_{i\min}$ is the minimum value of the corresponding score of the evaluation factor. The data sequence after dimensionless processing is $x_{i}=\left[x_{i}\left(1\right),x_{i}\left(2\right),\cdots ,x_{i}\left(m\right)\right],i=1,2,\cdots ,n$ .

Determining that the reference sequence is the maximum in the dimensionless data sequence, the reference sequence $x_{0}$ is x=0x01,x02,⋯,x0m .

The relational coefficient $\xi_{i\left(k\right)}$ between the number *k* element in $x_{0}$ and $x_{i}$ in the index layer is:


(9) $$\xi_{i\left(k\right)}=\frac{\begin{array}{l} \min i\min k|x_{0}\left(k\right)-x_{i}\left(k\right)|+\\ \rho \max i\max k|x_{0}\left(k\right)-x_{i}\left(k\right)| \end{array}}{\begin{array}{l} |x_{0}\left(k\right)-x_{i}\left(k\right)|+\rho \max i\\ \max k|x_{0}\left(k\right)-x_{i}\left(k\right)| \end{array}}$$

where *ρ* is the resolution coefficient, $\rho \in \left[0,\;1\right]$ , such that the smaller the ρ value, the greater the relational coefficient difference, and the stronger the ability to distinguish (usually ρ = 0.5); $\max i\max k|x_{0}\left(k\right)-x_{i}\left(k\right)|$ is the maximum value of the difference between the dimensionless data and the reference sequence; and $\min i\min k|x_{0}\left(k\right)-x_{i}\left(k\right)|$ is the minimum value of the difference between the dimensionless data and the reference sequence.

The relational coefficient of the criterion layer evaluation factor is the average value of the relational coefficient of the index layer evaluation factor. The relational coefficient of the criterion layer evaluation factor $\xi_{j}$ is:


(10) $$\xi_{j}=\frac{\xi_{i_{\left(k\right)}}+\xi_{i_{\left(k+1\right)}}+\cdots +\xi_{i_{\left(k+b-1\right)}}}{b}$$

where *b* is the number of the evaluation factors of the index layer under the criterion layer, $\xi_{i\left(k\right)}$ is the relational coefficient of the number *k* evaluation factor in the index layer, *k* = 1 + *b* × (*j* − 1) and $\xi_{j}$ is the relational coefficient of the number *j* evaluation factor in the criterion layer.

The grey relational degree $r_{0i}$ of the evaluation object calculated according to the relational coefficient of the index layer is:


(11) $$r_{0i}=\overset{m}{\underset{k=1}{\sum }}\omega_{k}\cdot \xi_{i\left(k\right)}$$

where $r_{0i}\in \left[0,1\right]$ , *m* is the number of evaluation factors in the index layer, $\omega_{k}$ is the weight of the number *k* evaluation factor, $\xi_{i\left(k\right)}$ is the relational coefficient of the number *k* evaluation factor and k=1,2,⋯,m . The greater the grey relational degree $r_{0i}$ , the higher the landscape level of the affiliated green space of the hospital.

### 2.5 Division of grade levels

According to the principles of GRA and in line with previous work (40), this study divided the hospital affiliated green spaces into five grades: grade 5, grey relational degree value ≥0.75; grade 4, grey relational degree value 0.65–0.75; grade 3, grey relational degree value 0.50–0.65; grade 2, grey relational degree value 0.30–0.50; and grade 1, grey relational degree value <0.30.

## 3 Results

### 3.1 Index weights

The evaluation system, which assigns between 1 and 5 points to each factor, was distributed to the target population, including experts in relevant professional fields, and the same number of patients in each of the three hospitals. Specific assignment and questionnaire questions can be found in the supplementary documents. The final sample comprised 100 respondents, giving a response rate of 93%. Basic information of the respondents is shown in Table 2.

**Table 2:** Basic information of the respondents.

Index		Male	Female
Number of people		52	48
Educational background	Primary school and below	16	17
	Junior middle school	23	19
	High school and above	13	12
Marriage situation	Married	23	21
	Unmarried	17	15
	Divorce	8	9
	Bereft of one’s spouse	4	3
The nature of one’s job	Brain work	27	28
	Manual labour	25	20
Residential address	City	29	26
	Village	23	22

According to Table 2, the number of 100 respondents, female and male, was 48 and 52, respectively, and the number of respondents with high school education or above was 25. Of the respondents, 55 lived in the city and 45 lived in the countryside. To understand the respondents’ assessment of patient recovery suitability in the hospital-affiliated green landscape, the study conducted data collection as a questionnaire. In the questionnaire, each index was evaluated in the form of scoring, and the highest score was 10 points. The higher the score, the higher the evaluation. Formulas 1–7 were used to process the data to determine the comprehensive weight of each level of the evaluation index (Table 3).

**Table 3:** Index weights for the evaluation system.

Target hierarchy (A)	Criterion layer (B)	Weight	Index layer (C)	Weight
Evaluation of patient recovery applicability of hospital affiliated green space landscape (A)	Plants (B1)	0.1628	Configuration of plants (C1)	0.0519
			Plant colours and seasonal change (C2)	0.0449
			Harmony between plants and the environment (C3)	0.0660
	Space (B2)	0.1978	Spatial diversity (C4)	0.0478
			Rationality of spatial scale (C5)	0.0780
			Rationalisation of spatial layout (C6)	0.0720
	Accessibility (B3)	0.3005	Comfort of facilities (C7)	0.0880
			Road safety (C8)	0.1293
			Information accessibility of facilities (C9)	0.0832
	Rehabilitation function (B4)	0.1709	Range of activities (C10)	0.0538
			Facilities participation (C11)	0.0595
			Caring landscape art (C12)	0.0576
	Publicity and education function (B5)	0.1680	Ornamental value (C13)	0.0602
			Degree of propaganda (C14)	0.0451
			Interactivity (C15)	0.0627

The evaluation index system consists of five evaluation factors in the criterion layer and 15 in the index layer. The factor weight for accessibility (B3) in the criterion layer is the highest (0.3005). And the factor weight for plants (B1) is the lowest (0.1628). Landscape accessibility plays a vital role in the criterion layer as a basic need of the target population. Hospital patients are the largest beneficiary group, because the accessibility of the landscape is essential for them. Plants occupy a complementary position in the rehabilitation landscape. Plant morphological collocations, seasonal changes and coordination with the environment stand in subtle relations to human physiology and psychology, and it takes a long time for them to achieve beneficial effects on people’s bodies and minds.

In the index layer, road safety (C8) has the highest weight (0.1293), and plant colours and seasonal change (C2) have the lowest weight (0.0449). Road safety therefore occupies an important position in the indicator layer. The security risks of landscape roads directly affect the safety of users and the accessibility of landscapes. Thus, the security of landscape roads is fundamental if patients are to visit and to undergo rehabilitation there. The category of plant colours and seasonal change includes the morphological changes of plants over the phenological period of a year, which represents a long cycle. In contrast, visits to the hospital affiliated green space are of no fixed duration; the tour cycle is short, and little attention is paid to the landscape changes of the hospital green space over the year.

EWM was used to calculate the index weights, using data from the 93 valid questionnaires to ensure the accuracy of the calculations. The data are accurate and reliable, and the evaluation system is reasonable, allowing the applicability of hospital affiliated green spaces to patient recovery to be evaluated.

### 3.2 Comprehensive evaluation

According to the relevant principles of the GRA method, the grey relational coefficient of the index layer factor was calculated using Formula 9 (Table 4).

**Table 4:** Grey relational coefficients for the index layer.

Index layer factor	Second Hospital	Tumour Hospital	Fifth Hospital
Plant configuration (C1)	1.0000	0.3333	0.5188
Plant colours and seasonal change (C2)	1.0000	0.3333	0.4915
Harmony between plants and the environment (C3)	1.0000	0.3333	0.3448
Spatial diversity (C4)	1.0000	0.3333	0.3491
Rationality of spatial scale (C5)	1.0000	0.6358	0.3333
Rationalisation of spatial layout (C6)	1.0000	0.6195	0.3333
Comfort of facilities (C7)	1.0000	0.3333	0.4224
Road safety (C8)	0.8479	1.0000	0.3333
Information accessibility of facilities (C9)	0.5133	0.3333	1.0000
Range of activities (C10)	1.0000	0.4367	0.3333
Facilities participation (C11)	0.4511	0.3333	1.0000
Caring landscape art (C12)	1.0000	0.6009	0.3333
Ornamental value (C13)	1.0000	0.4009	0.3333
Degree of propaganda (C14)	0.4293	0.3333	1.0000
Interactivity (C15)	0.5389	1.0000	0.3333

According to the GRA results for the index layer factor, the grey relational coefficients of the criterion layer factor were calculated using Formula 10 (Table 5).

**Table 5:** Grey relational coefficients for the criterion layer.

Criterion layer factor	Second Hospital	Tumour Hospital	Fifth Hospital
Plants (B1)	1.0000	0.3333	0.4517
Space (B2)	1.0000	0.5295	0.3386
Accessibility (B3)	0.7871	0.5556	0.5853
Rehabilitation function (B4)	0.8170	0.4570	0.5556
Publicity and education function (B5)	0.6561	0.5781	0.5556

**Figure 2:**
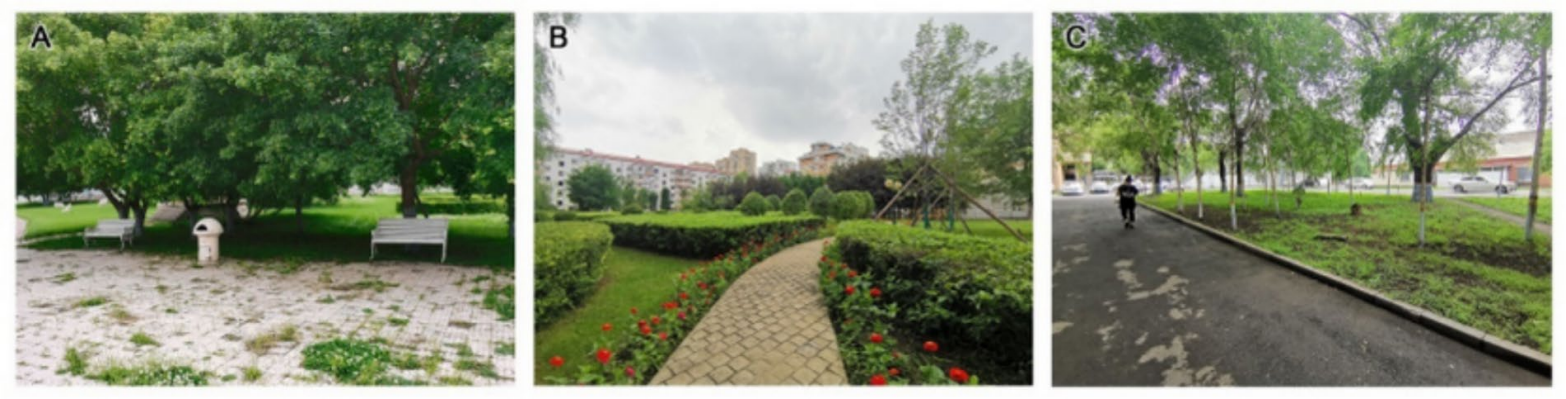
Plant configurations at **(A)** Second Hospsital, **(B)** Tumour Hospital, and **(C)** Fifth Hospital.

According to the evaluation results reported in Tables 3, 4, the grey relational coefficient (ξj) for plants (B1) at Second Hospital was highest (1.0000). Fifth Hospital was in second place (0.4517), and Tumour Hospital had the lowest coefficient (0.3333). Second Hospital has clear advantages in plant configuration, plant colours and seasonal changes, and plant and environmental coordination. In terms of plant configuration (C1), compared with Tumour Hospital and Fifth Hospital, Second Hospital has a larger green area, more plant species, a more abundant vertical greening layer and a reasonable plant collocation (Figure 2A). Shrubs dominate the green space at Tumour Hospital, where tall trees are scarce (Figure 2B). In contrast, tall trees dominate the Fifth Hospital’s green space, with less ground cover and shrubs, resulting in a relatively open field of view under the tree cover (Figure 2C).

**Figure 3:**
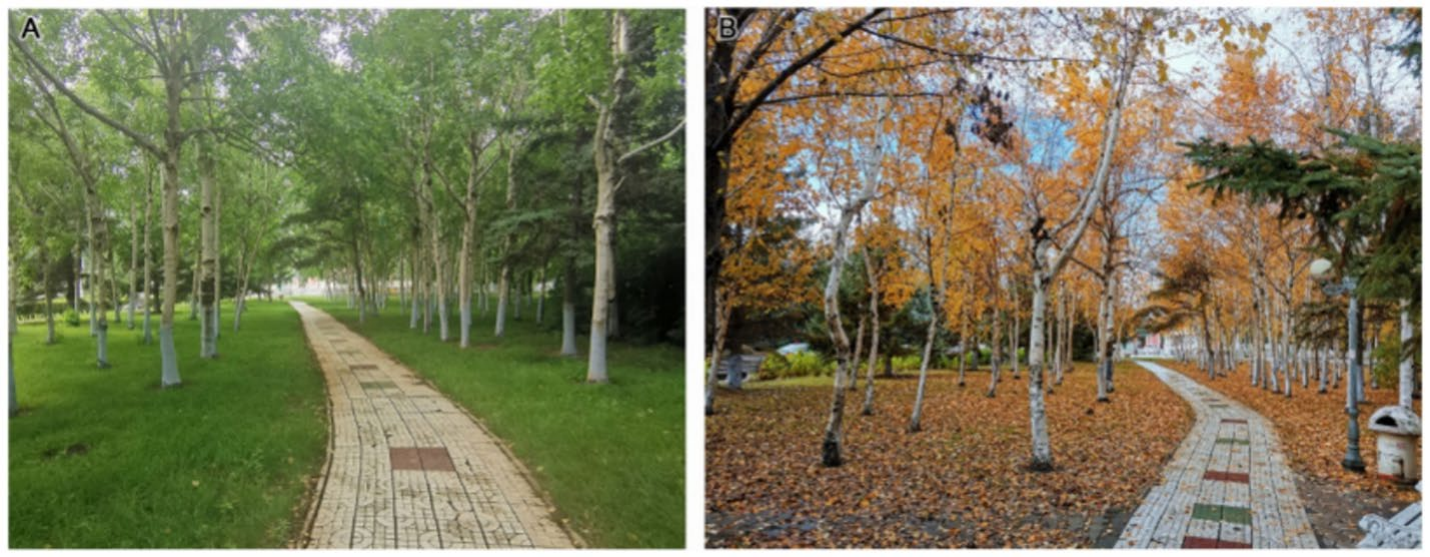
Plant landscape at Second Hospital **(A)** in summer and **(B)** in autumn.

In terms of plant colours and seasonal changes (C2), Second Hospital is rich in plant species, with trees in four seasons and flowers in three seasons (Figure 3). The plant configurations of Fifth Hospital and Tumour Hospital focus on trees and/or shrubs, resulting in an open field of view under the tree cover, a monotonous edge line and the insufficient spatial level of the canopy line.

**Figure 4:**
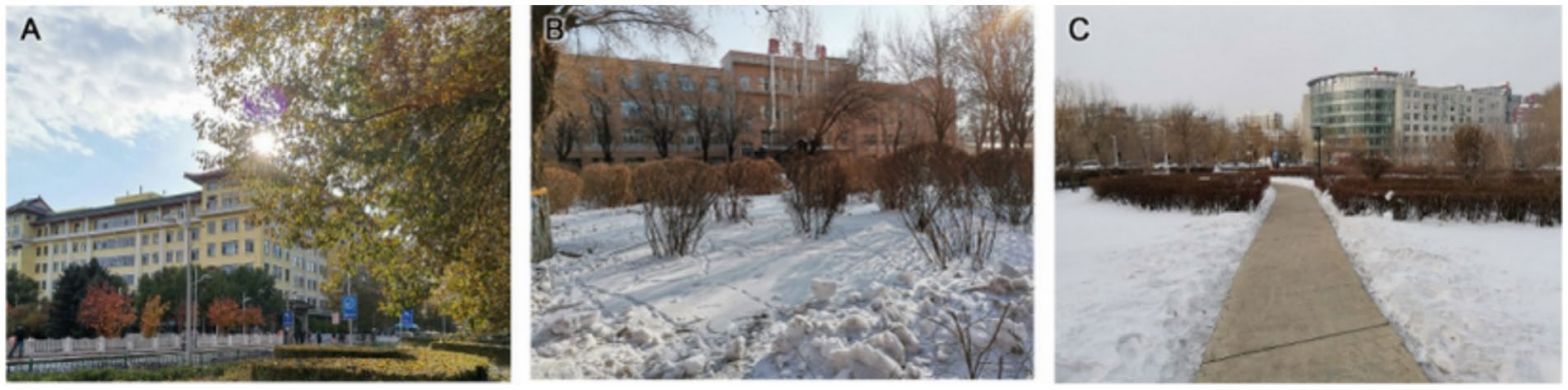
The building and landscape of **(A)** Second Hospital, **(B)** Fifth Hospital, and **(C)** Tumour Hospital.

In terms of harmony between plants and the environment (C3), the green space of Second Hospital is better coordinated with the surrounding buildings. The plant design softens the outline of the building, reduces its abruptness and creates good landscape continuity. The green spaces of Fifth Hospital and Tumour Hospital play a role in shielding the surrounding buildings from view, but the landscape effect is not good (Figure 4). Richer plant species, more diverse colour matching and a distinct aspect would provide better plant landscape conditions for the hospital’s rehabilitation green space, and these aspects should be considered carefully in landscape design.

For space (B2), the highest grey relational coefficient (ξj) was that of Second Hospital (1.0000), with Tumour Hospital in second place (0.5295) and Fifth Hospital had the lowest coefficient (0.3386). Second Hospital has absolute advantages in terms of spatial diversity, scale and layout. In terms of spatial diversity (C4), compared with those at Fifth Hospital and Tumour Hospital, the space types in the landscape at Second Hospital are richer, the spatial functions are more complete and the changes in the vertical landscape generate a sense of hierarchy. The spatial scale rationality (C5) of Second Hospital and Tumour Hospital are more appropriate than that of Fifth Hospital, in that they better guarantee the safety and convenience of users. Regarding rationality of spatial layout (C6), the road system at Second Hospital is excellent, as the park road covers an appropriate distance and is well coordinated with the surrounding space. The designers of Tumour Hospital used symmetry and balance to connect the main road with the green landscape spaces on the east and west sides, thus creating greater interest for people visiting the space. The combination of road and space also enhances the overall sense of coordination of the green landscape. In contrast, Fifth Hospital uses steps to divide the functional space; as a result, the sense of boundary between the spaces is not strong, and the layout is not reasonable (41).

**Figure 5:**
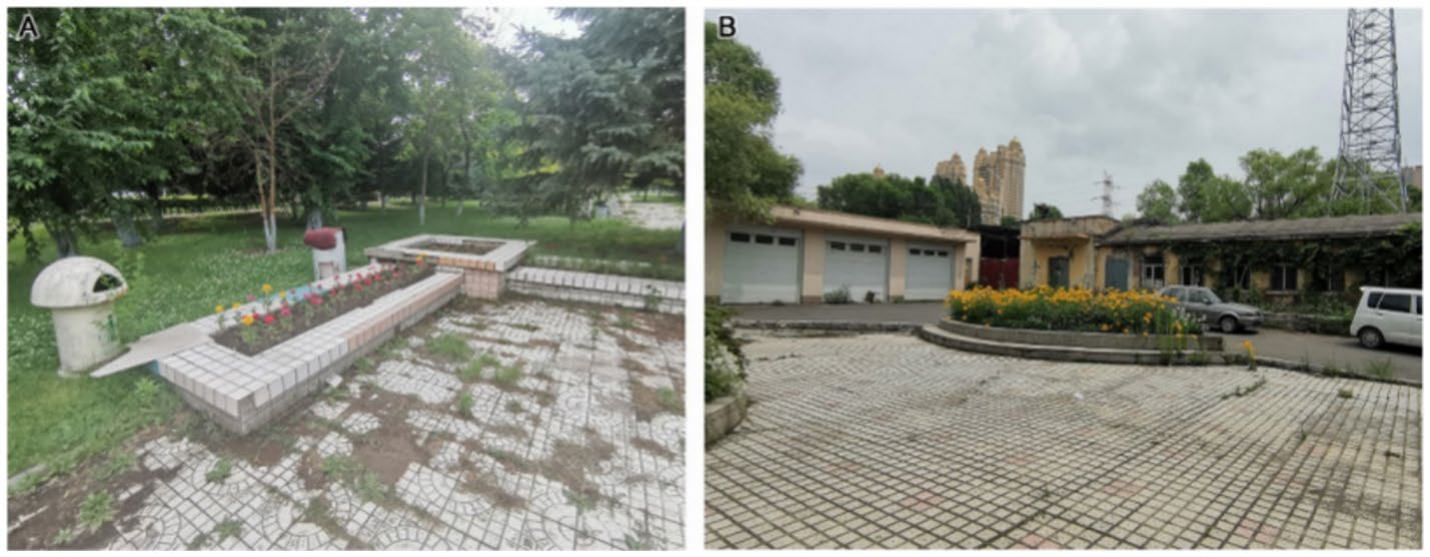
Low flower pools at **(A)** Second Hospital and **(B)** Fifth Hospital.

**Figure 6:**
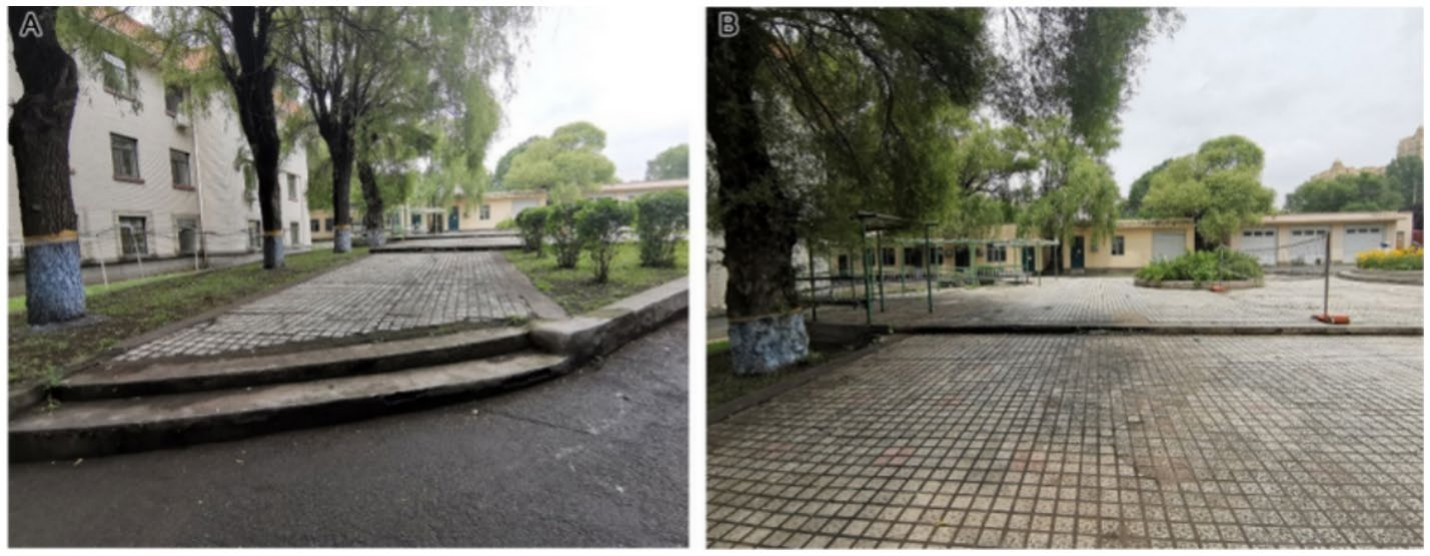
Fifth Hospital: **(A)** height difference treatment and **(B)** badminton courts.

For accessibility (B3), the grey relational coefficient (ξj) of Second Hospital was highest (0.7871), followed by Fifth Hospital (0.5853) and then Tumour Hospital (0.5556). All three hospitals perform well in relation to the comfort of their facilities, road safety and the accessibility of information about the facilities. In terms of comfort (C7), Second Hospital had the highest correlation value. Field investigation showed that Second Hospital and Fifth Hospital have low flower pools (Figure 5), which is highly suitable, but that none of the three hospitals provides armrests for patients to lean on during visits. In general, the facilities of the three hospitals are deficient in scale, material and artistic sense. In terms of road safety (C8), although Tumour Hospital had the highest correlation value, the roads of all three hospitals are free of obstacles such as cable poles. The overall terrain at Tumour Hospital and Second Hospital is flat, and the height difference is mainly managed with ramps. The garden road follows a barrier-free design, and the road safety factor is high. The main element in the height difference management at Fifth Hospital is steps (Figure 6A), creating potential safety hazards for patients who use wheelchairs or suffer from movement disorders. In terms of facility information accessibility (C9), Fifth Hospital had the highest correlation value. The hospital has clear guidance signs at the side of the road; these signs are unshielded, easy to identify and provide a strong sense of direction. The landscape facilities have explanation boards that convey information in a straightforward way, allowing users to find out what they need to know at a glance. Landscape accessibility is fundamental to ensuring the function of landscape rehabilitation, and an excellent barrier-free landscape level plays a significant role in increasing the rehabilitation value of hospital affiliated green space.

**Figure 7:**
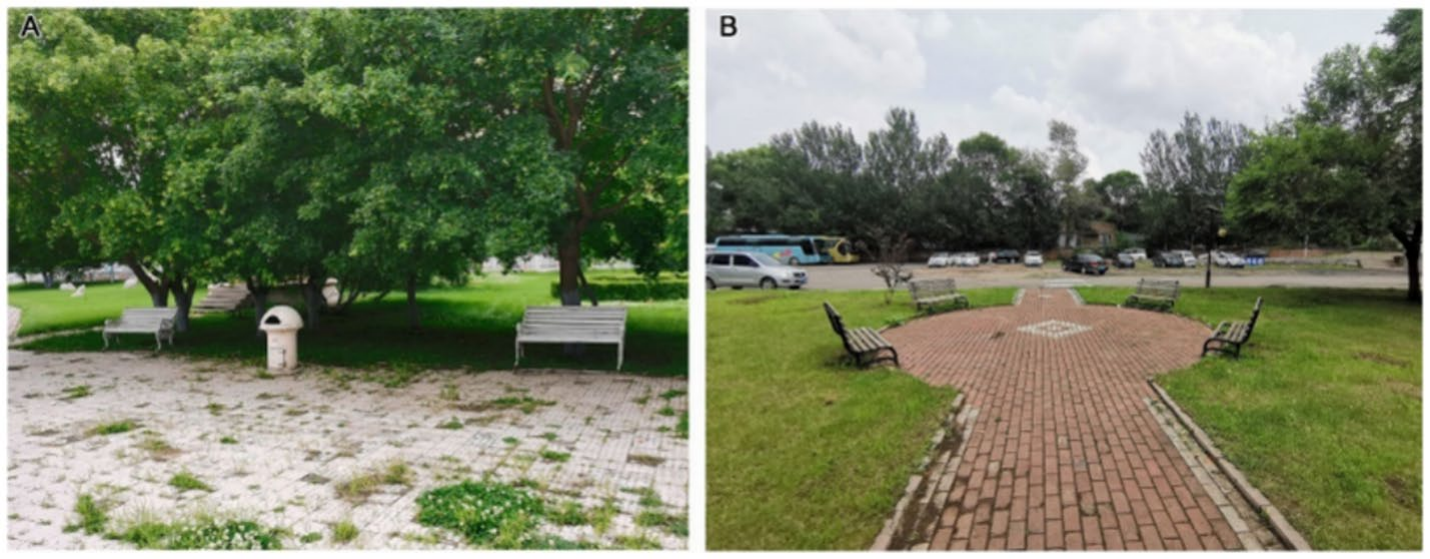
Leisure seats at **(A)** Second Hospital and **(B)** Tumour Hospital.

The grey relational coefficient (ξj) for rehabilitation function (B4) was highest at Second Hospital (0.8170), followed by Fifth Hospital (0.5556) and then Tumour Hospital (0.4570). Second Hospital performs well in its range of activities and landscape art care, whereas Fifth Hospital performs well in facility participation. In terms of the diversity of functional rehabilitation activities (C10), Second Hospital had the highest correlation value. Both Second Hospital and Tumour Hospital have leisure squares, where recovering patients can take part in activities such as Tai Chi and dance. The squares lend themselves to different types of activities, which is conducive to the physical rehabilitation of patients who can exercise independently. In contrast, Fifth Hospital has set up a single-activity space, namely a badminton court (Figure 6B). However, the level of participation in rehabilitation facilities also determines the degree of the positive effect of affiliated green space on patients’ physical rehabilitation, and it is Fifth Hospital that had the highest correlation value for facility participation (C11). By setting up landscape facilities such as badminton courts and leisure corridors, Fifth Hospital allows recovering patients to participate in activities and to rest. The inclusiveness of participation is wide, which has a great positive impact on patients’ physical rehabilitation. In contrast, the landscape facilities of Second Hospital and Tumour Hospital are mainly leisure seats (Figure 7); this type of rehabilitation facility is insufficient, and improvements are necessary.

**Figure 8:**
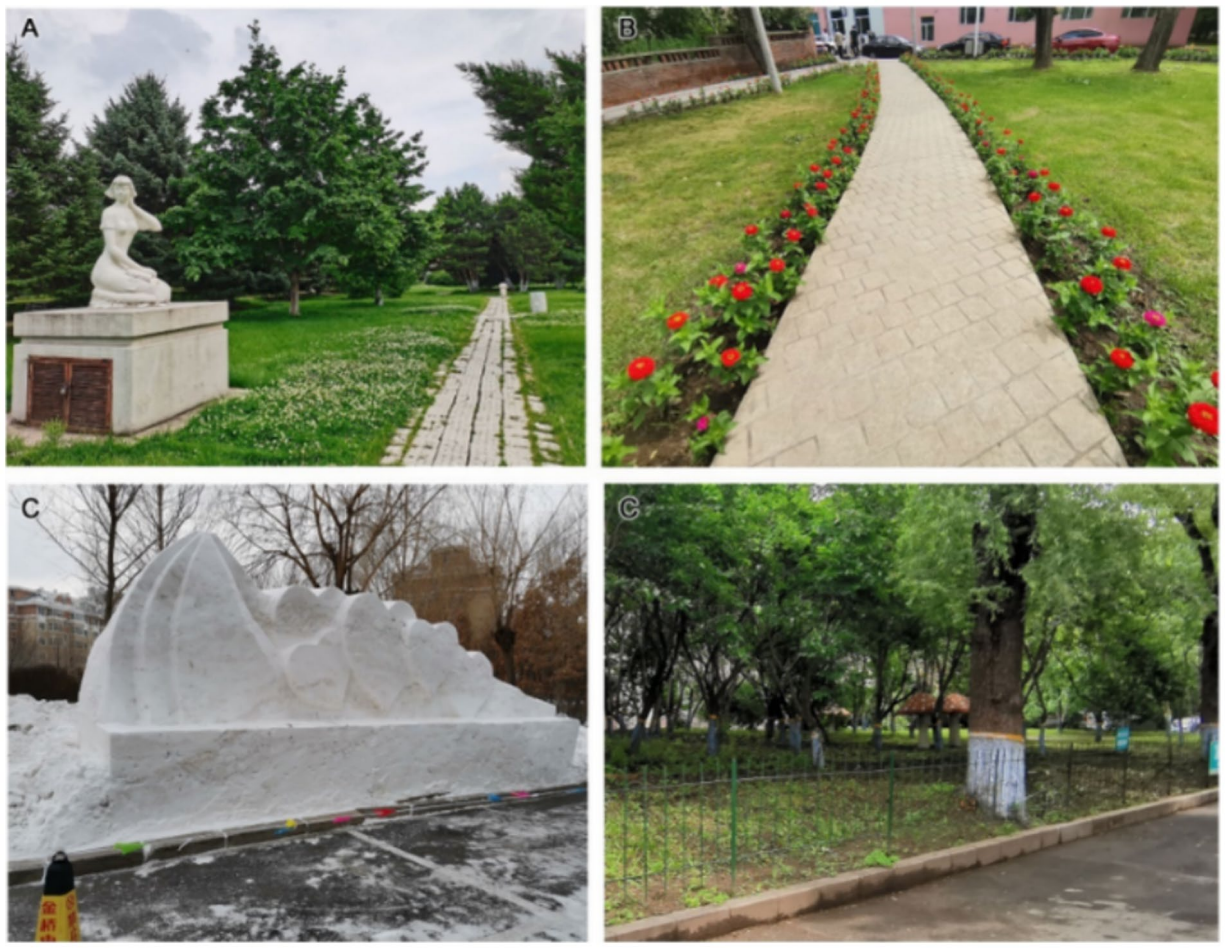
Landscape art care at the three hospitals: **(A)** figure sculptures at Second Hospital; **(B)** flower borders at Tumour Hospital; **(C)** snow sculpture at Tumour Hospital; **(D)** mushroom sculptures at Fifth Hospital.

For landscape art care (C12), Second Hospital had the highest correlation value, reflecting its richness in terms of trees, shrubs and grass, as well as its commemorative figure sculptures (Figure 8A) for people to observe and admire. The space is full of artistic sense in respect of form and colour, which encourages patients’ physical and mental well-being and plays a positive role in rehabilitation treatment at the psychological level. The green space area at Tumour Hospital is large, and the distribution site is composed of flowering plants and rest seats. In summer, colourful flower borders are planted at the side of the path (Figure 8B); in winter, artistic snow sculptures are set up (Figure 8C). Thus, the targeted design of the landscape uses seasonal changes to produce psychological changes in patients. The affiliated green space of Fifth Hospital, however, provides only a few mushroom sculptures. The sense of interaction is weak (Figure 8D), and the artistic sense and care level of the landscape needs to be improved. The healing function of landscape is an important attribute of hospital affiliated green spaces, and this analysis shows that the three hospitals under study have given inadequate consideration for the diversity of rehabilitation functions and the hierarchical goals of the service population in green space landscape design. It is difficult to provide good rehabilitation conditions for patients during recovery, and the design of green spaces should focus on the needs of the target population, combine landscape and function and increase the diversity of rehabilitation activities.

**Figure 9:**
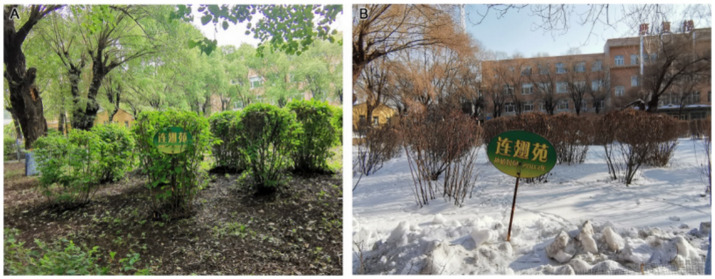
Plant commentary board at Fifth Hospital **(A)** in summer and **(B)** in autumn.

For publicity and education (B5), the grey relational coefficient (ξj) was highest for Second Hospital (0.6561), followed by Tumour Hospital (0.5781) and then Fifth Hospital (0.5556). Each of the three hospitals has advantages in terms of landscape appreciation, publicity and interaction. For publicity and education landscape ornamental value (C13), Second Hospital had the highest correlation value. Its commemorative figure sculptures, like the winter snow sculpture of Tumour Hospital, perform well with regard to science popularisation, educational sense and artistic appreciation. In contrast, the publicity and education form of the Fifth Hospital landscape is primarily reflected in the plant commentary board (Figure 9), and there is less interaction with tourists. The colour of the card is similar to that of the plants, which is not convenient for summer identification, and the artistic sense is poor. According to the questionnaire survey results, 86.7% of the respondents were satisfied with the landscape sculpture in the hospital, while only 5.8% were dissatisfied with this survey.

**Figure 10:**
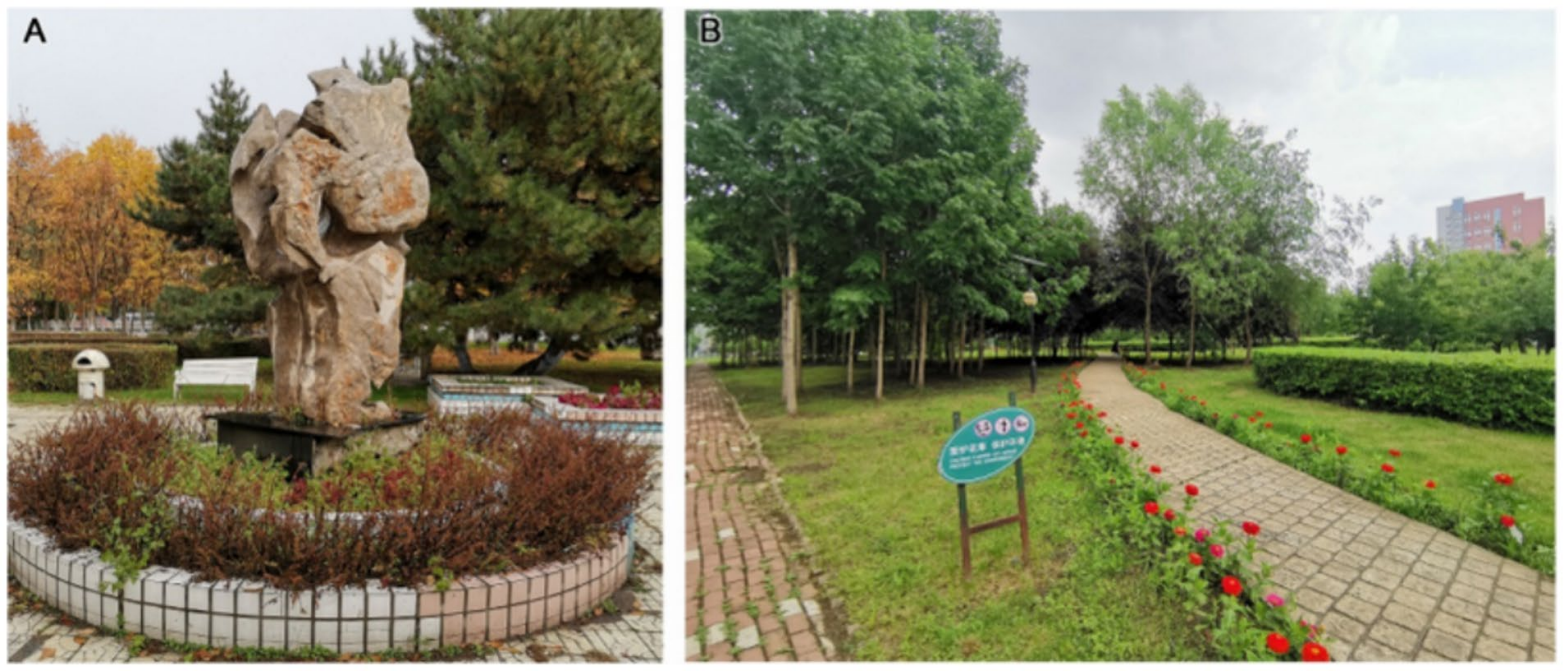
Publicity and education landscapes: **(A)** abstract art sculptures at Second Hospital and **(B)** banners at Tumour Hospital.

For publicity and education landscape propaganda (C14), Fifth Hospital had the highest correlation value. Although its main means of landscape propaganda and education are the plant interpretation boards, which have a single function and low artistry, the degree of information transmission is high. In this category, Second Hospital relies primarily on commemorative figures and abstract art sculptures (Figure 10A), with a single form but high artistic appreciation. Tumour Hospital has set up banners (Figure 10B) to announce slogans to visitors. In terms of the interactivity of propaganda and the education landscape (C15), Tumour Hospital had the highest correlation value. Compared with the plant branding at Fifth Hospital, the winter snow sculpture at Tumour Hospital and the characters and abstract sculpture at Second Hospital fulfil their propaganda and education functions better. The landscape modelling is also more interesting; because it attracts tourists to interact with it, it can enhance the effects of propaganda and education. Hospitals should increase the intensity of their science publicity, so that patients visiting the green space can benefit from knowledge and pleasure, which promote rehabilitation.

Based on the grey relational coefficients for the index layer factor, the grey relational degree (r_0i_) for the landscape and rehabilitation of the affiliated green spaces in the three hospitals was calculated using Formula 11 (Table 6).

**Table 6:** Grey relational degrees.

Hospital	Grey relational degree (r_0i_)
Second Hospital	0.8525
Tumour Hospital	0.5306
Fifth Hospital	0.4846

Table 6 and the grade level divisions show that the grey correlation degree (r_0i_) of Second Hospital was the highest (0.8525, grade 5), followed by Tumour Hospital (0.5306, grade 3) and then Fifth Hospital (0.4846, grade 2). The patient recovery applicability of the landscape at Second Hospital is currently high, whereas Tumour Hospital and Fifth Hospital have considerable room for improvement and development. On-the-spot investigation indicates that the green plant landscape is good at all three hospitals, but that there are shortcomings in the expression of the propaganda and education landscape. To some extent, these shortcomings reflect inadequacies in the construction of the green space environment as a healing landscape, in that the medical environment lacks a combination of ‘soft and hard’ features. Hospital affiliated green space should follow the principle of ‘suitable scale, perfect function and leisure’, integrating design, rehabilitation and education functions, increasing outdoor activities and designing roads and facilities in line with a happiness scale.

## 4 Discussion

This study formulated evaluation indexes according to the basic requirements of landscape construction (including plant configuration and spatial layout) and the needs of target groups (such as landscape rehabilitation and propaganda and education functions). EWM was used to calculate objectively the comprehensive weights of the evaluation indexes, and GRA was used to evaluate multiple target landscapes. The results were then analysed in detail applying rigorous mathematical logic. The evaluation system is reasonable and an appropriate mathematical method was selected to ensure that the calculation results were as accurate as possible.

In terms of evaluation indicators, most previous studies have selected indicators based on the requirements of landscape construction and user comfort. However, more consideration is necessary for the care of the hospital landscape and the availability of information; the form and intensity of propaganda and education are not enough. In terms of mathematical models, most previous studies have used AHP to calculate the index weights (29–32). Compared with EWM, AHP is more subjective. In terms of the selection of target sites, some studies have focused on single-site landscapes only, and their results are correspondingly weak in relation to horizontal comparison and correlations with similar landscapes. In contrast, the present study took the applicability of landscape to patient recovery as its focus, established an evaluation system, created a targeted evaluation and applied reasonable evaluation criteria. It used the relatively objective EWM to calculate the index weights, and it selected three hospitals with a large green area so that the patient recovery applicability evaluation indexes could be analysed both horizontally and vertically, in a rigorous, reasonable and representative manner.

The results show that the affiliated green spaces of the three hospitals have different advantages and disadvantages in relation to the 15 evaluation indexes. Overall, Second Hospital had the highest evaluation, with Tumour Hospital in second place and Fifth Hospital had the lowest evaluation. At present, the patient recovery applicability of landscape in Second Hospital is high, whereas both Tumour Hospital and Fifth Hospital have considerable room for improvement. Second Hospital pays attention to landscape accessibility and care, and the quality of its popular science landscape is good; nonetheless, its propaganda content is inadequate. Tumour Hospital focuses on the psychological healing of patients in environmental and spiritual aspects and pays attention to the accessibility of the landscape; however, it is lacking in propaganda and education science. Fifth Hospital’s education content is limited to a single form, and the aesthetics of its landscape signs and its consideration of landscape barrier-free design are inadequate.

There are five main practical implications of the findings of this study. (1) In terms of plant landscape, all three hospitals attach importance to plant landscape design, but with a different focus. Second Hospital focuses on the matching of plant species and levels. Its vertical greening landscape is rich in levels, and the colour matching and seasonal changes of the plants are good. Tumour Hospital mainly uses low shrubs and flowers, with few tall trees; the spatial level of the plant landscape is thus not rich, and the edge line of the wooded area is monotonous. Fifth Hospital is dominated by tall trees, and the view under the wooded area is relatively open. (2) In terms of space, the three hospitals have different space divisions and layouts. Second Hospital uses the plant enclosure space, and the functional area division is clear. Tumour Hospital takes the main road as the main axis and uses symmetry around the central axis to define the spatial layout; the good fit with the road space increases landscape coordination. Fifth Hospital mainly uses steps to divide the space. Its road system lacks guidance and warning signs, as well as visual and tactile stimulation, which increases the risk to patients visiting. (3) In terms of accessibility, the treatment methods of the three hospitals have advantages and disadvantages. The main advantage is that the overall terrain is relatively flat, which allows the garden road to follow a barrier-free design. The main disadvantage is that the details have not been considered carefully enough. The garden road at Fifth Hospital uses steps to deal with the elevation difference, which increases movement difficulties for patients in rehabilitation. The low-level flower pools of Second Hospital and Fifth Hospital do not provide handrails on which patients can rest. At Tumour Hospital, even less overall consideration has been given to barrier-free design. (4) In terms of rehabilitation function, all three hospitals focus on physical rehabilitation and psychological healing. The shortcomings are that the types of targeted rehabilitation facilities provided are insufficient, and that the patient recovery applicability of the landscape has room for improvement. Second Hospital has a leisure square and art sculptures; although its rehabilitation facilities are relatively popular, they provide only a single form of propaganda and education. Tumour Hospital shapes its flower landscape in summer and sets out art snow carvings in winter, but it provides psychological healing for patients only from the environmental and spiritual aspects, to the neglect of rehabilitation facilities. Fifth Hospital offers a badminton court, a leisure gallery and simple sculptures, but it provides less functional space for rehabilitation and rehabilitation facilities, and its facilities for rehabilitation and psychological healing are poor. (5) In terms of publicity and education, there is room for improvement at all three hospitals. Second Hospital focuses on commemorative sculptures with poor artistic sense. The signs in the green space of Tumour Hospital are not visually attractive. Fifth Hospital’s sole form of propaganda and education, namely its plant commentary cards, are excellent for popularisation but are limited to a single function and characterised by low artistry. Overall, the three hospitals have advantages and disadvantages across the evaluation indicators. The main shortcomings are that the accessibility of the landscape is inadequate, the range of targeted rehabilitation facilities is narrow, little importance is accorded to propaganda and education in the landscape, and the applicability of landscape rehabilitation in the three hospitals could be improved.

In summary, this study shows that the focus in hospital affiliated green space construction should be on maintaining a good green environment, achieving a reasonable spatial layout, improving road safety, ensuring a range of facilities and taking account of science education publicity. In terms of plants, designers should pay attention to the hierarchy of trees, shrubs and grass, the expression of the landscape over four seasons, the use of plants to divide dynamic and static spaces and the integration of plants into the surrounding environment. It is crucial to use plants to soften architectural contours and mitigate any sense of abruptness, so that the landscape has a certain continuity. In terms of spatial layout, designers can divide spaces using pavement patterns, colours and materials (instead of, for example, pedestal seats), thereby achieving both visual and tactile transition or segmentation of space. In terms of accessibility, important measures include regular monitoring of road safety, designs that handle elevation differences effectively, provision of eye-catching signs at necessary nodes and ensuring the safety of users by enabling them to obtain relevant information directly through specific composition or strong colour contrast. In terms of rehabilitation function, the focus should be on the basic needs of the target population: increasing the diversity and pertinence of rehabilitation activities (24, 25), handling the sense of scale of rehabilitation facilities, minimising safety hazards, increasing humanistic care and refining the local culture (42, 43). Disease-prevention measures and spiritual aspects must also be integrated into landscape nodes. In terms of publicity and science education, the integration of a range of technical means and landscapes will increase users’ interest in publicity, thus enriching the forms of expression of propaganda and the education landscape. The three hospitals under study already perform well in terms of treatment equipment (the hard environment), but the rehabilitation value of their affiliated green spaces (the soft environment) is inadequate. Excellent hospital affiliated green space can increase the pleasure and knowledge that the target population derives from the landscape, thereby serving patients better in each period of their recovery. To promote the development of healing landscapes, the government should provide active guidance on rehabilitation landscapes, increase investment in construction and establish a sound database for rehabilitation science alongside a comprehensive and relevant evaluation system.

## 5 Conclusion

The rehabilitation function of hospital green space as an indirect medical means is an important trend in relation to healing resources in the medical spaces of the future. The integration of landscape and rehabilitation should be explored in detail to provide the target population with functional plots that are convenient to visit and that can fulfil their rehabilitation functions. The scientific evaluation system proposed in this study can be used as a basis for improving the design of hospital healing landscapes. It covers five aspects: plants, space, accessibility, rehabilitation function and publicity and education function.

Starting from the requirements of landscape construction and user satisfaction, the system was used to evaluate the patient recovery applicability of the affiliated green spaces of three hospitals in Harbin, and the results of the evaluation were compared horizontally and vertically. The grey relational degree of Second Hospital was highest (0.8525, grade 5), followed by Tumour Hospital (0.5306, grade 3) and then Fifth Hospital (0.4846, grade 2). The grey relational coefficient for plants (B1) and space (B2) at Second Hospital was relatively high, whereas the coefficients for accessibility (B3), rehabilitation function (B4) and publicity and education function (B5) of all three hospitals were low.

Drawing on problem-oriented analysis of the results, this study has proposed optimisation strategies to improve the function of hospital affiliated green spaces, such as regular monitoring of road safety, an increase in targeted activity facilities, greater attention to humanistic care and improvements in relation to propaganda and popularisation of science. Adoption of the recommended strategies will lead to greater humanisation of the hospital landscape rehabilitation function system and will benefit the general public. The research methods used in this paper to carry out rapid and effective evaluation of the patient recovery applicability of hospital green spaces can also be applied to the evaluation of other landscape environments. This study studied the rehabilitation function of hospital-affiliated green spaces from a macro perspective. It aimed to comprehensively and accurately explain the current situation regarding the landscape rehabilitation applicability of hospital-affiliated green spaces, and lay the foundation for transforming these spaces. In short, the main design elements of the hospital affiliated green space should include hierarchical vegetation, road patterns, rich colors, accessibility facilities and rehabilitation facilities. Among them, the establishment of rehabilitation facilities in the green space is helpful to meet the spiritual needs of patients and improve their enthusiasm for rehabilitation. However, there remains a need for in-depth research on some specific healing indicators of hospital-affiliated green spaces, such as indicators regarding the healing function of certain plant species (e.g., flowers, leaves, fragrant plants, plants of different colours, etc.), road classification from a professional landscape design perspective, and consideration of the proportion of water features, landscape design details that are most conducive to patient rehabilitation, and the reasons and mechanisms underlying the healing function of rehabilitation gardens.

## Data availability statement

The original contributions presented in the study are included in the article/supplementary material, further inquiries can be directed to the corresponding author.

## Author contributions

WY: Writing – original draft, Formal analysis, Conceptualization. MY: Writing – original draft, Methodology. DL: Writing – original draft, Methodology, Formal analysis, Data curation. XF: Writing – review & editing, Methodology, Formal analysis. LY: Writing – review & editing, Methodology. KW: Writing – review & editing, Conceptualization.
